# The M, F and HN genes of genotype VIId Newcastle disease virus are associated with the severe pathological changes in the spleen of chickens

**DOI:** 10.1186/s12985-015-0366-5

**Published:** 2015-09-04

**Authors:** Yan Kai, Zenglei Hu, Haixu Xu, Shunlin Hu, Jie Zhu, Jiao Hu, Xiaoquan Wang, Xiaowen Liu, Xiufan Liu

**Affiliations:** Animal Infectious Disease Laboratory, College of Veterinary Medicine, Yangzhou University, 12 East Wenhui Road, Yangzhou, Jiangsu Province 225009 China; Jiangsu Co-innovation Center for Prevention and Control of Important Animal Infectious Diseases and Zoonoses, Yangzhou, Jiangsu China

## Abstract

**Background:**

The strains of the genotype VIId Newcastle disease virus (NDV) induce more severe tissue damage in lymphoid organs than other virulent strains. The underlying molecular mechanisms are poorly understood.

**Methods:**

Genotype IV NDV Herts/33 and genotype VIId NDV JS5/05 have a distinctive pathological profile in the spleen. These two strains of viruses were selected as parental viruses to generate a panel of chimeric viruses by replacing the M, F and HN genes of Herts/33 individually or in combination with the corresponding genes of JS5/05 using reverse genetic. Virulence and *in vitro* characteristics of the recombinant viruses were assessed. In addition, pathological changes, virus load, and transcriptional cytokine response in the spleen of chickens infected with these recombinant viruses were also analyzed.

**Results:**

Pathogenicity test showed that all chimeric viruses are virulent. *In vitro* characterization revealed that gene replacement did not change growth kinetics and HN expression on cell surface of the recombinant viruses. However, replacement of the M, F and HN genes resulted in apparent changes in the fusion activity. Moreover, pathological studies revealed that only inclusion of the homologous M, F and HN genes of JS5/05 in Herts/33 backbone resulted in severe pathological changes characterized by extensive necrosis in the spleen, similar to that induced by JS5/05. In addition, this gene replacement significantly increased virus replication and the levels of transcriptional cytokine response, compared to Herts/33. Conversely, inclusion of the M, F and HN genes of Herts/33 into JS5/05 backbone resulted in Herts/33-specific pathological changes and significantly decreased virus load and the expression levels of cytokine genes, compared to JS5/05.

**Conclusions:**

The M, F and HN genes are related to the severe pathological changes in the spleen of chickens infected with genotype VIId NDV.

## Background

Newcastle disease virus (NDV) is an important pathogen threatening the poultry industry. Based on the disease severity in chickens, NDV strains are divided into three pathotypes: lentogenic, mesogenic and velogenic [1]. It is generally accepted that the cleavage site in the fusion (F) protein is the primary virulence determinant of NDV [2, 3]. NDV tropism mainly depends on the virus pathotype, and generally viscerotropic velogenic strains cause severe lesions in the lymphoid system, intestinal tract and respiratory tract. However, different virulent NDV strains sharing similar F cleavage site associated with high virulence induce distinct pathological manifestation in chickens, especially in lymphoid tissues [4–7]. Some recent studies have shown that some NDV isolates of genotype VIId induce more severe tissue damage in lymphoid organs compared to other virulent NDV strains [5, 6, 8]. In addition, we have provided both *in vitro* and *in vivo* data that high levels of virus replication and an intense inflammatory response contribute to this pathological manifestation of genotype VIId of NDV [8, 9].

Since NDV is an evolving pathogen and has a high genetic diversity, it is necessary to determine the molecular basis for the severe pathological changes in the lymphoid organs caused by genotype VIId of NDV. Our previous studies demonstrate that genotype VIId NDV strain JS5/05 and genotype IV NDV strain Herts/33 induce contrasting pathological manifestation in lymphoid tissues in chickens [6, 8]. JS5/05 induces extensive necrosis and marked lymphocyte depletion in the spleen, whereas Herts/33 only induces atrophy and mild to moderate lymphoid depletion [6, 8]. In addition, strains of genotype VII and IV are evolutionally divergent and the genetic homology between JS5/05 and Herts/33 whole genome is 23.4 %. We aim to determine the molecular basis of the severe pathology in lymphoid tissues caused by genotype VII NDV using reverse genetics. In this study, the matrix (M), F and hemagglutinin-neuraminidase (HN) genes of Herts/33 were replaced with the corresponding genes from JS5/05 individually or in combination. Pathology, virus replication and cytokine response in the spleen induced by the recombinant viruses were compared.

## Results

### Rescue of Herts/33 and the chimeric viruses

**Fig. 1:**
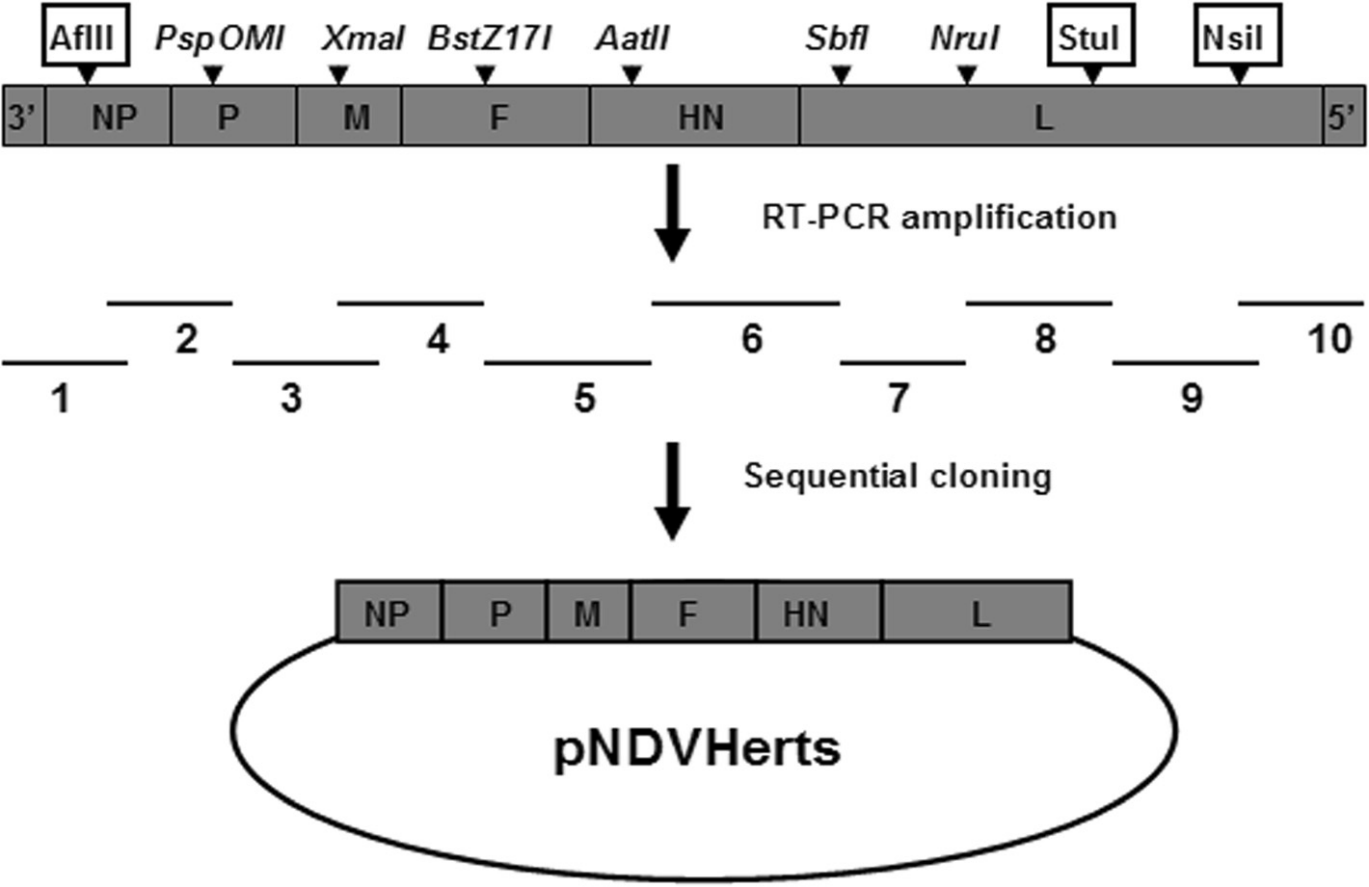
Schematic representation of the clone strategy for the full-length antigenomic cDNA clone of Herts/33. Ten overlapped cDNA fragments spanning the entire genome were generated and cloned into the transcription vector TVT (0.0). Six unique restriction sites (italicized) and three additional sites (boxed) were used to sequentially assemble these fragments

**Fig. 2:**
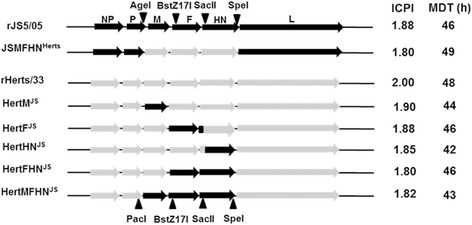
Gene map illustrating gene replacement strategy in Herts/33 backbone and pathogenicity of the recombinant viruses. Three unique restriction sites in the JS5/05 genome and the PacI in the Herts/33 genome were used to replace viral genes. Intracerebral pathogenicity index (ICPI) and mean death time (MDT) were measured to evaluate the pathogenicity of the chimeric viruses. For designation of the chimeric viruses, Herts/33 backbone is shown in short as Hert and the donator virus JS5/05 is shown as superscript

The infectious clone of Herts/33 was constructed following the cloning strategy illustrated in Fig. 1. Infectious rHerts/33 virus was successfully rescued by co-transfection of the Herts/33 full-length clone together with three ZJ1-derived helper plasmids into BSR-T7/5 cells. The hemagglutination (HA) titer of the rescued virus was 8 log2, similar to that of the wild-type strain. Sequencing of the rHerts/33 virus confirmed the lack of mutation or recombination during virus rescue in the presence of the heterologous supporting plasmids. In addition, gene replacement between JS5/05 and Herts/33 backbones was conducted as shown in Fig. 2. All viable recombinant viruses were rescued using NDV reverse genetic system and showed comparable HA titer as the parental viruses (data not shown), indicating the genetic compatibility between JS5/05 and Herts/33.

### Virulence of the recombinant viruses

Intracerebral pathogenicity index (ICPI) and mean death time (MDT) in eggs were measured to evaluate the virulence of the recombinant viruses. We found that ICPI values of all chimeric viruses were above 1.80, and MDT values were lower than 60 h (Fig. 2), which are comparable to that of the parental strain, suggesting that all recombinant viruses have high virulence.

### *In vitro* characterization of the recombinant viruses

**Fig. 3:**
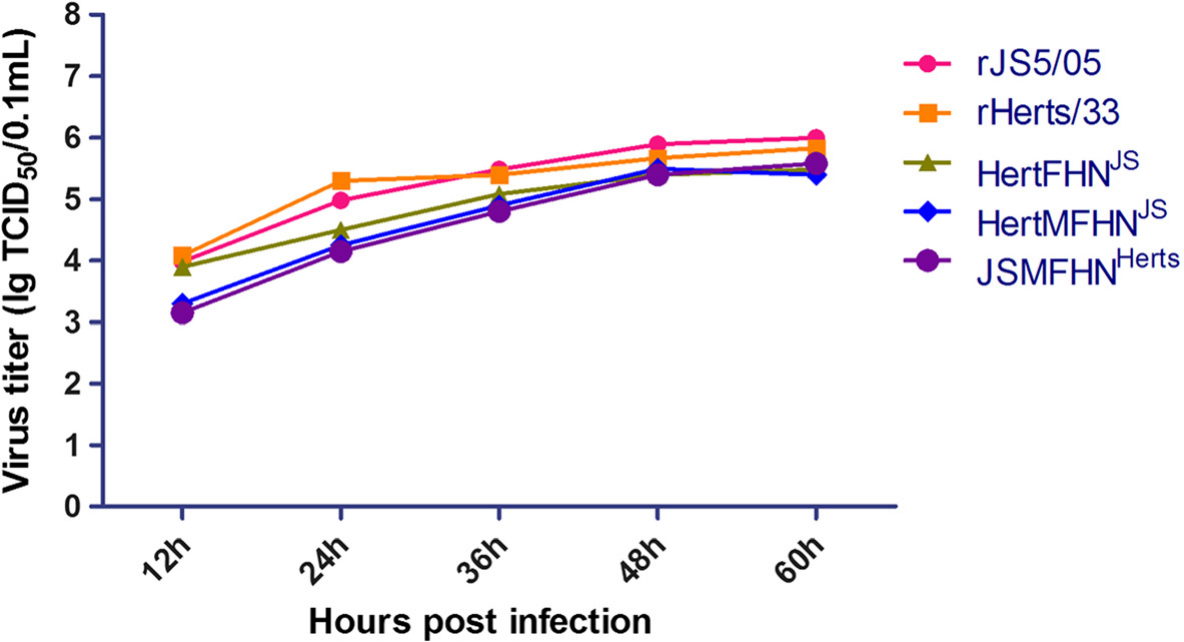
Growth curves of the viruses. The growth kinetics of the parental and recombinant viruses was assessed by a multistep growth assay in DF1 cells. Each virus was inoculated at MOI of 0.001 into DF1 cells and the cell supernatant was collected at every 12 h until 60 h p.i. for titration

Based on the virus titers of the collected samples, the growth curves of the viruses were obtained. As shown in the Fig. 3, the similar growth kinetics was observed between the rHerts/33 and rJS5/05 and the recombinant viruses showed lower propagation compared with the parental strains at 12 and 24 h post-inoculation (p.i.). However, all the viruses had similar virus titers after 36 h p.i.

**Fig. 4:**
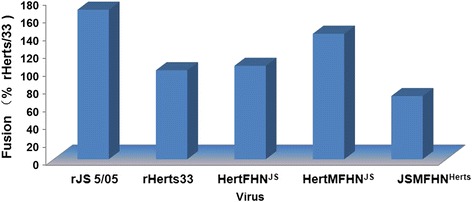
Fusion activity of the viruses. The fusion ability of the recombinant viruses was examined in DF1 cells infected at MOI of 0.001. The cells were stained with Giemsa for syncytium observation. The fusion activity was expressed as a percentage of rHerts/33 whose fusion activity was considered as 100 %

Regarding the fusion activity of the viruses, JS5/05 and two Herts/33-based recombinant viruses showed stronger fusion ability than rHerts/33 (Fig. 4). Inclusion of the M, F and HN genes of JS5/05 into Herts/33 backbone resulted in a marked increase of the fusion ability, and vice versa (Fig. 4). These data suggest that the M, F and HN genes might be associated with the higher cell fusion ability of JS5/05.

### Quantitation of HN expression on cell surface

**Fig. 5:**
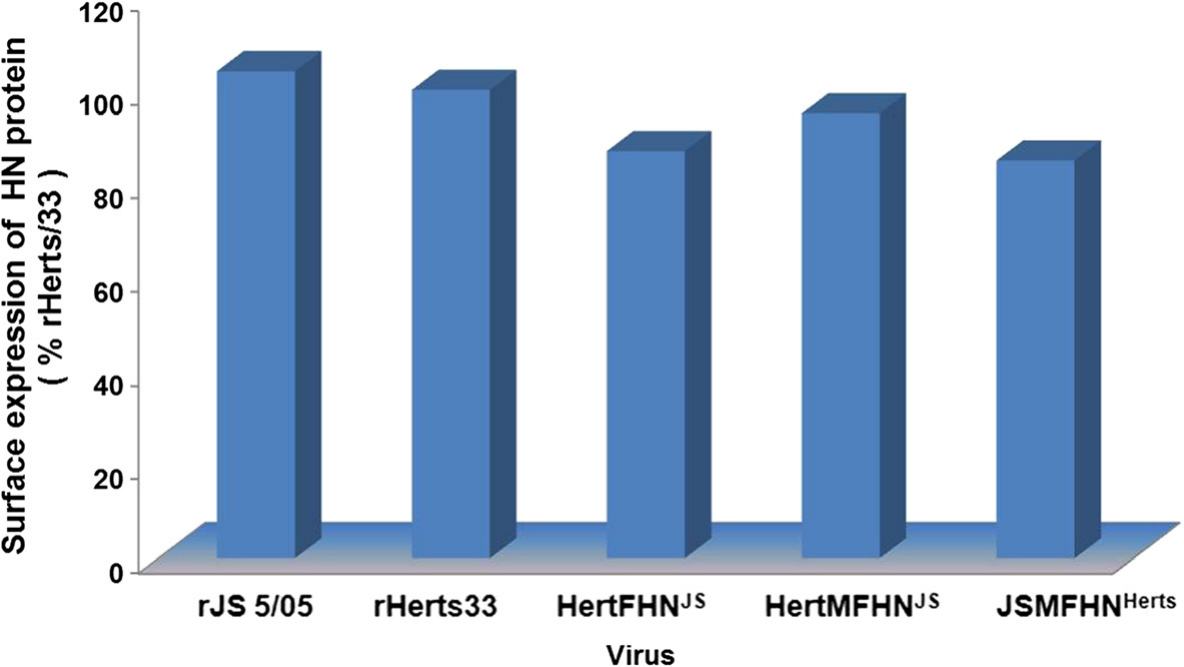
Expression quantity of HN protein on cell surface. The cell surface expression of the glycoprotein HN was determined by the FACS analysis. The DF1 cells infected at MOI of 0.001 were incubated with mAb 6B1 and goat anti-mouse IgG-FITC in turn. The HN expression level was reflected by the fluorescence intensity compared to the parental virus rHerts/33

The cell surface expression of the glycoprotein HN was determined by the FACS analysis (Fig. 5). The data showed there was no significant difference in HN expression in the infected cells between the two parental viruses. Gene replacement led to slight decrease in HN expression in infected cells compared to the corresponding parental virus, but no significant difference was observed. These results showed that gene replacement did not affect HN expression on cell surface of the recombinant viruses.

### Clinicopathological assessment of the chimeric viruses

Since JS5/05 and Herts/33 induce remarkably contrasting pathological changes in the spleen, the splenic lesion was used as the primary parameter to evaluate the pathogenic role of viral genes. We found that all tested viruses killed all chickens within 5 days p.i., indicating that they are highly pathogenic to chickens, and that gene replacement does not decrease the pathogenicity of the parental virus in chickens.

**Fig. 6:**
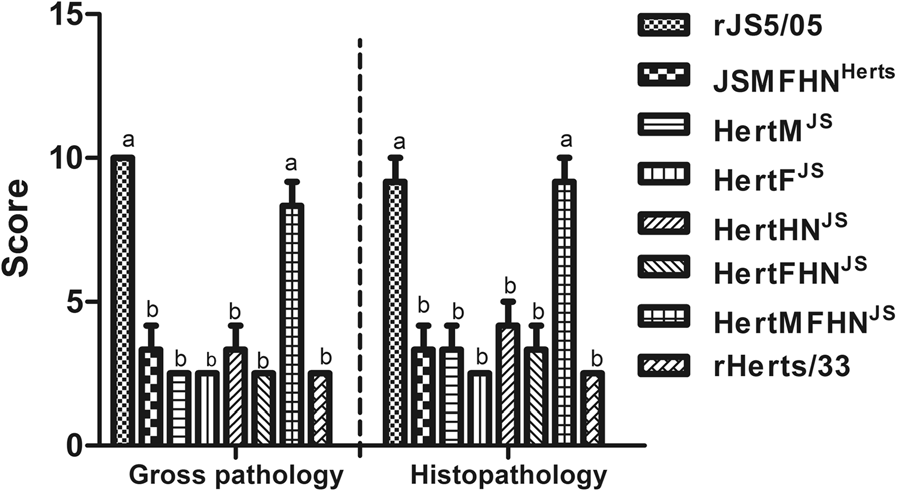
Scoring of pathological changes. Gross and histologic changes in the spleen were scored based on the following standards: Gross lesion (0 for normal; 2.5 for atrophy without necrosis; 5 for enlarged and mottled; 7.5 for mild to moderate necrotic foci; 10 for extensive and severe necrosis); histologic changes (0 for normal; 2.5 for mild to moderate hyperplasia of lymphocytes; 5 for mild lymphocyte depletion; 7.5 for moderate (<50 %) lymphocyte depletion, histiocytic accumulation and necrosis; 10 for severe (>50 %) lymphocyte depletion, and extensive necrosis0. Average scores for three chickens were presented. Data were analyzed using One-way ANOVA followed by LSD multiple-comparison test. Lowercase letters on top of each stand for statistical difference, different letters mean a significant difference between two groups

Gross examination showed that chickens infected with rJS5/05 had enlarged spleens with widespread foci of necrosis both on the capsular and cut surfaces, whereas rHerts/33-infected birds had atrophic spleens without necrotic lesions, which is consistent with the previous findings [6, 8]. With respect to the recombinant viruses, inclusion of the single JS5/05 HN, F or M gene into the Herts/33 backbone (HertHN^JS^, HertF^JS^ and HertM^JS^) resulted in the Herts/33-specific pathological changes characterized by splenic atrophy and lack of necrosis. Inclusion of both the F and HN genes of JS5/05 into the Herts/33 backbone was also unable to induce JS5/05-specific necrotic lesions. More importantly, chickens infected with HertMFHN^JS^ displayed severe pathological changes in the spleen characterized by remarkable necrotic lesions, resembling that produced by rJS5/05. Conversely, simultaneous inclusion of the M, F and HN genes of Herts/33 into JS5/05 backbone led to the elimination of the necrotic foci. Scoring of gross pathological changes showed that only inclusion of the M, F and HN genes of JS5/05 significantly increased the severity of gross lesions in the spleen compared to rHerts/33, and simultaneous inclusion of the M, F and HN genes of Herts/33 in to JS5/05 backbone significantly decreased the severity of splenic damage (Fig. 6).

**Fig. 7:**
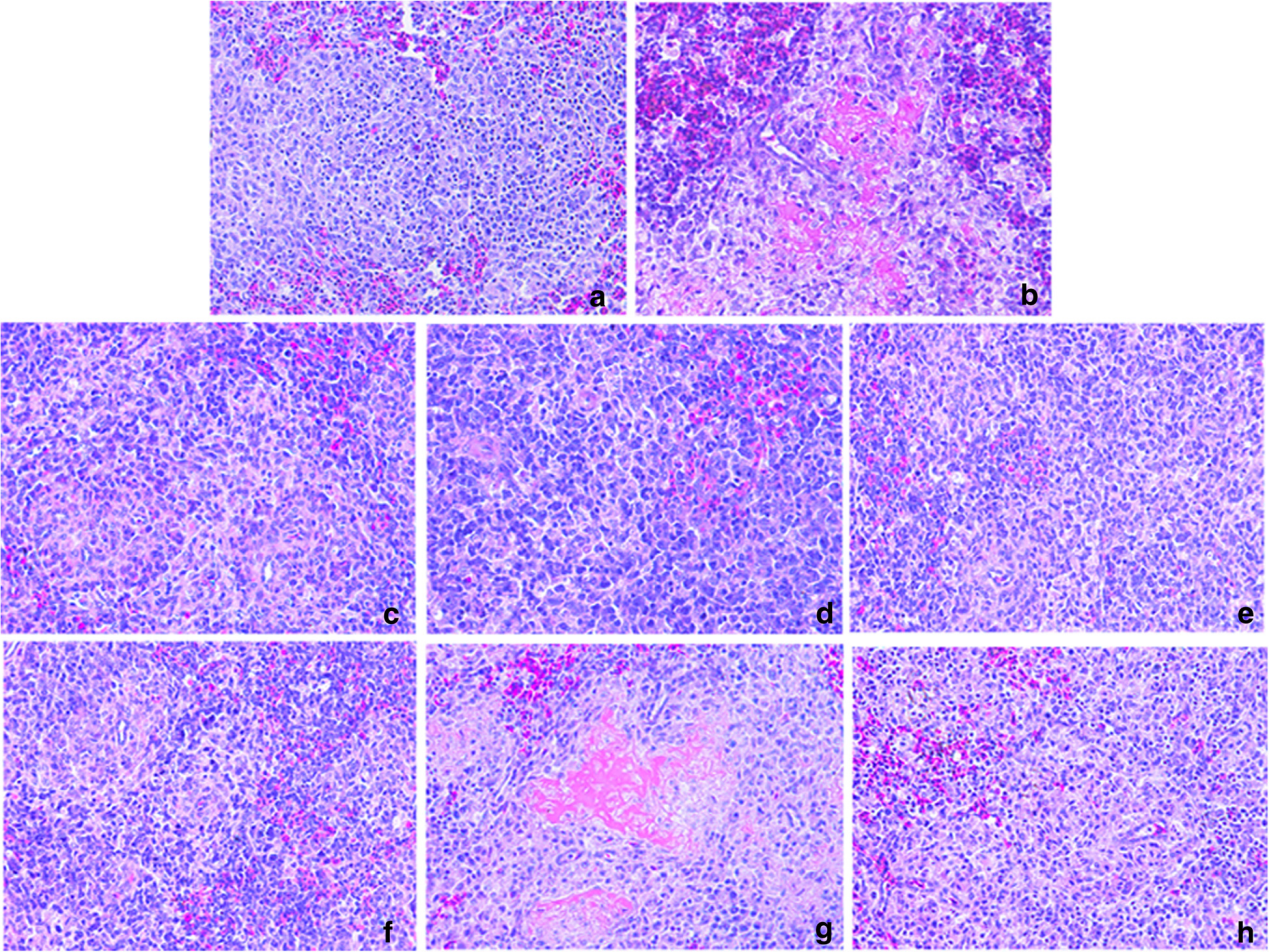
Histological changes in the spleen caused by the chimeric viruses. JSMFHN^Herts^ (**a**), HertHN^JS^ (**c**), HertF^JS^ (**d**), HertM^JS^ (**e**) and HertFHN^JS^ (**f**) induced mild to moderate lymphocyte depletion and infiltration, similar to that caused by rHerts/33 (**h**). HertMFHN^JS^ (**g**) caused severe splenic necrosis, similar to that induced by rJS5/05 (**b**). Magnification, ×200

rJS5/05 produced the most remarkable histological lesions in the spleen, including multifocal to coalescing necrosis, marked lymphocyte depletion and extensive deposits of fibrins following necrosis (Fig. 7b). In contrast, only mild to moderate hyperplasia or depletion of lymphocytes were observed in birds infected with rHerts/33 (Fig. 7h). Mild to moderate lymphocyte depletion and moderate infiltration of macrophages in the spleens of chickens infected with HertHN^JS^, HertF^JS^ and HertM^JS^ and HertFHN^JS^ (Fig. 7c-f) were observed, similar to that of rHerts/33. However, additional inclusion of the M gene of JS5/05 into HertFHN^JS^ resulted in severe necrotic lesions and extensive lymphocyte depletion (Fig. 7g) and this manifestation was largely ablated in rJS5/05 by simultaneous replacement of the M, F and HN genes of Herts/33 (Fig. 7a). In addition, histological changes scoring revealed that among all gene replacements, only the inclusion of the M, F and HN genes of JS5/05 into Herts/33 backbone significantly enhanced the extent of tissue damage in the spleen (Fig. 6). These results demonstrated that pathological manifestation in the spleen induced by rJS5/05 and rHerts/33 depended on the origin of the M, F and HN genes.

### Virus replication

**Fig. 8:**
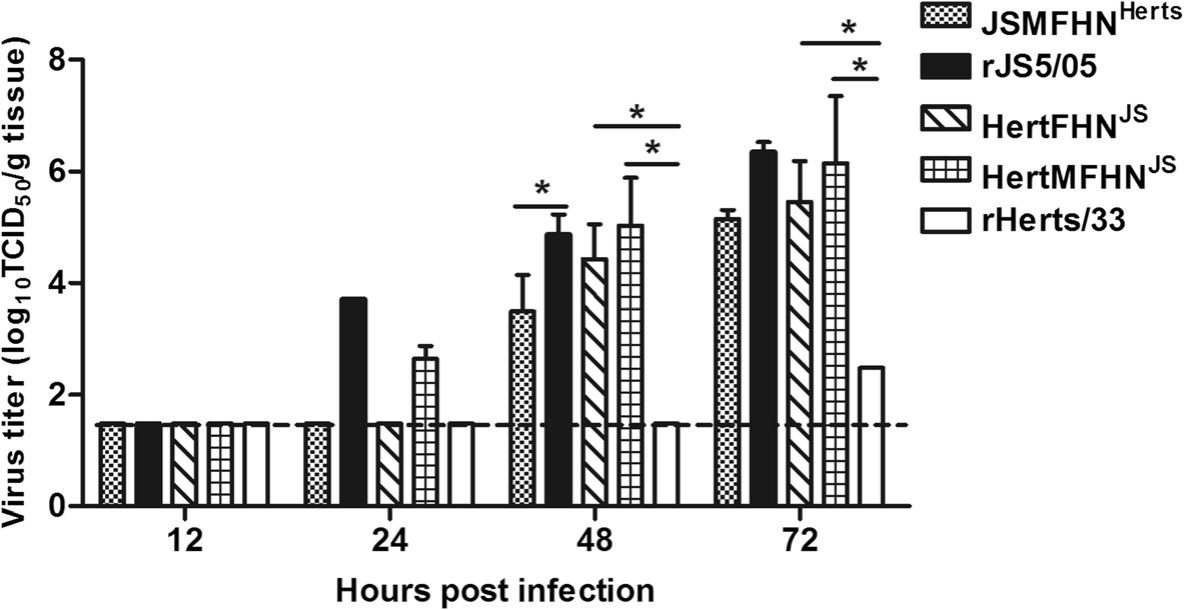
Viral load of the chimeric viruses in the spleen. Tissue virus loads were determined in chicken embryo fibroblasts and expressed as the average mean titers (log10 TCID50/g tissue). Error bars show standard error of the mean (SEM). Virus titers were analyzed among the chimeric viruses derived from the same backbone using one-way ANOVA followed by LSD multiple-comparison test. Asterisk (*) indicates significant difference at *p* < 0.05. The dotted lines indicate the lower limit of virus detection

Since pathological assessment showed that the M, F and HN genes play a major role in spleen damage caused by the JS5/05 virus, the replication rate of recombinant viruses with three replaced genes in the spleen was analyzed. Additionally, recombinant Herts/33 carrying the F and HN genes of JS5/05 was also included due to the importance of homotypic F and HN proteins for NDV pathogenicity. As shown in Fig. 8, only rJS5/05 and HertMFHN^JS^ established an early infection at 24 h p.i. in the spleen among a panel of recombinant viruses. At 48 h p.i., virus titer of rHerts/33 was below the detection limit, and inclusion of both the F and HN genes of JS5/05 increased the rate of Herts/33 virus replication. Additional inclusion of the M gene of JS5/05 further elevated virus replication. Conversely, simultaneous inclusion of the M, F and HN genes of Herts/33 in JS5/05 backbone significantly reduced virus load in the spleen. Similar trend of virus replication was observed at 72 h p.i. These results suggest that the M, F and HN genes are associated with high level of replication of JS5/05 in the spleen.

### Transcriptional cytokine response

**Fig. 9:**
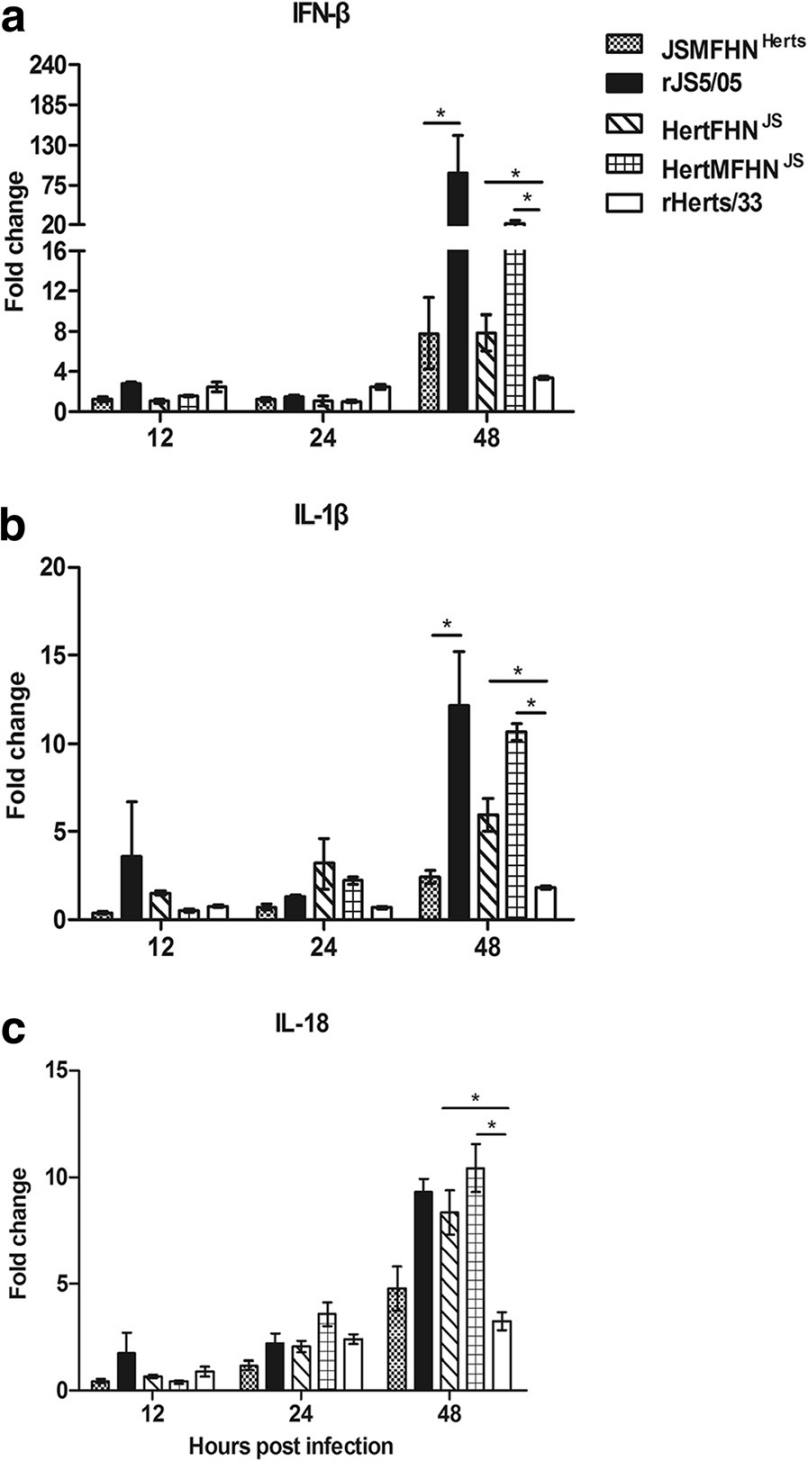
RT-qPCR analysis of cytokine genes in the spleen of chickens infected with the chimeric viruses. **a** IFN-β. **b** IL-1β. **c** IL-18. The data are the mean fold change ± standard error of the mean (SEM). Expression values of cytokine genes were compared among the chimeric viruses sharing the same backbone using one-way ANOVA followed by LSD multiple-comparison test. Asterisk (*) indicates significant difference at *p* < 0.05

The host innate immune response to NDV is an important contributor to NDV pathology. According to the previous data [8], we selected interferon (IFN)-β, interleukin (IL)-1β and IL-18 representing different branches of the innate immune response. The transcriptional levels of these cytokine genes were measured in the spleens of chickens infected with the chimeric viruses. As shown in Fig. 9, the expression levels of IFN-β, IL-1β and IL-18 in the spleens of chickens infected with any recombinant virus were low at 12 and 24 h p.i. However, at 48 h p.i., inclusion of the F and HN genes of JS5/05 in Herts/33 backbone significantly enhanced the expression of the tested cytokine genes, compared to the parental virus rHerts/33. Additional inclusion of the homologous M gene further increased the expression levels of cytokine genes. Conversely, three-way introduction of the M, F and HN genes of Herts/33 into JS5/05 backbone resulted in significant decrease in the expression levels of cytokine genes when compared to rJS5/05. Taken together, these findings suggest that the M, F and HN genes are responsible for the high level transcriptional activities of cytokines in JS5/05 virus-infected chickens.

## Discussion

Prior studies have shown that some NDV isolates of genotype VIId induce more severe damage in lymphoid tissues than other virulent strains. However, the molecular determinant for this phenomenon is not clear. In this study, we demonstrated that, when the M, F and HN genes of genotype IV Herts/33 were replaced with corresponding genes from genotype VIId JS5/05, the recombinant virus caused JS5/05 virus-like necrosis in the spleen. In addition, this recombinant Herts/33 virus led to a significantly higher replication rate and a stronger innate immune response than the parental virus. Our data suggest that the M, F and HN genes of NDV genotype VIId are responsible for the severe pathologic change in the spleen.

In order to understand the molecular basis of the severe pathologic changes caused by genotype VIId NDV, JS5/05 and Herts/33 were selected because of their distinct pathological potency in the spleen [6, 8]. These two viruses belong to two distant phylogenic genotypes, and the nucleotide homologies of the M, F and HN genes between Herts/33 and JS5/05 are low M: 87.4 %; F: 87.7 %; HN: 85.6 %). Interestingly, our results showed that virulence (ICPI and MDT) (Fig. 2) and pathogenicity in the chickens infected with the Herts/33-derived recombinant viruses was similar to that infected with rHerts/33, suggesting that the genes of JS5/05 virus are compatible in Herts/33 backbone. Moreover, *in vitro* characterization of the recombinant viruses, including the growth kinetics, the fusion activity and HN presentation on cell surface, demonstrated that gene replacement did not change these biological features of the recombinant viruses (Figs. 3, 4 and 5).

Since pathological changes in the spleen are the main characteristics that distinguish two parental viruses, gross and pathological changes in the spleen were used as the primary parameters to evaluate the pathogenic role of viral genes. Our study revealed that inclusion of the single M, F and HN gene as well as both the F and HN genes of JS5/05 into Herts/33 failed to produce JS5/05-specific necrotic lesions in the spleen, but simultaneous replacement of the M, F and HN genes resulted in severe necrosis and lymphocyte depletion, resembling that of rJS5/05. Conversely, inclusion of the M, F and HN genes of Herts/33 together into JS5/05 backbone led to the elimination of JS5/05-specific necrotic lesions (Figs. 6 and 7). These findings indicate that the M, F and HN genes of JS5/05 are associated with the severe splenic pathogenicity. We further demonstrated that the expression of the M, F and HN genes from JS5/05 in Herts/33 backbone significantly increased the rate of virus replication (Fig. 8) and the transcriptional levels of cytokine genes (Fig. 9) in the spleen, compared to rHerts/33. It is worthwhile to point out that the recombinant JS5/05 carrying the M, F and HN genes of Herts/33 displayed higher replication rate than rHerts/33 in the spleen at 48 and 72 h p.i, indicating that the M, F and HN genes of Herts/33 don’t decrease virus replication of the chimeric virus to the level of Herts/33. It is possible that the interaction between the three envelop-associated proteins of Herts/33 and the internal proteins of JS5/05 is not optimal for virus replication. However, there is no significant difference in the transcriptional activity of all tested cytokine genes between JSMFHN^Herts^ and rHerts/33. These findings indicate that virus replication and the innate immune response simultaneously determine the severity of the splenic damage. This is consistent with our previous data that a high virus replication rate and strong innate immune response contribute to the severe pathology caused by genotype VIId NDV strains in lymphoid tissues [8].

It is believed that homologous cytoplasmic tails (CT) of the F and HN proteins of paramyxovirus are critical for their interaction. The homotypic F and HN interaction is important for paramyxiviruses for proper function of these proteins [10–13]. In our study, the replacement of the single F and HN genes was incomplete since the homologous CT domains were left intact during gene replacement (Fig. 2). Thus, the interactions between these two glycoproteins may be not impaired. Only the presence of the homologous M, F and HN genes may retain the optimal interactions between viral proteins, which may explain the largely conversion of the pathology phenotype observed for HertMFHN^JS^ and JSMFHN^Herts^.

Using a backbone virus to replace various genes from other virus strains, several studies have identified the homotypic F and HN genes as key players in NDV virulence [13–15]. We also observed that inclusion of both F and HN genes of JS5/05 into Herts/33 backbone led to a significant increase in virus replication and cytokine response. These data are consistent with the findings from Cornax et al. that the F and HN genes from velogenic strain CA02 markedly improve the replication of mesogenic strain Anhinga/93 in macrophages [16]. However, the modification of Herts/33 with JS5/05 F and HN genes did not lead to the pathological changes seen in JS5/05-infected chickens. It is possible that the replacement of both F and HN genes is not sufficient to change pathology of typical velogenic NDV, and the M gene cooperates with F and HN genes to achieve the maximal damage in lymphoid tissues.

Recently, Paldurai et al. [17] reported a comprehensive study on NDV pathogenicity through replacing individual viral genes or in different combination between velogenic strain Texas GB and mesogenic strain Beaudette C. The F protein is the major contributor for virulence, the homologous M and HN proteins play a supporting role [17]. Although we did not perform gene swap between the Herts/33 and JS5/05 strains, we made similar conclusions that the presence of three envelop-associated proteins determines certain phenotypes of NDV. However, in addition to pathology or virulence, other features of NDV, such as incorporation of heterotypic glycoproteins in to the chimeric virions and the interaction between the three envelop-associated proteins with heterotypic internal proteins, deserve more studies.

## Conclusions

Our study showed that the M, F and HN genes are associated with the high level of virus replication and intense innate immune response of genotype VIId NDV strain JS5/05. Increased virus load and strong innate immunity of JS5/05 strain may contribute to the severe pathologic changes in the spleen. Our study narrows down the pathogenicity of genotype VIId NDV virus into these three genes, laying a foundation for further fine-mapping the epitopes responsible for NDV pathogenicity.

## Methods

### Ethics statement

Animal experiments were conducted according to the guidelines of Jiangsu laboratory animal welfare and ethics of Jiangsu Administrative Committee of Laboratory Animals and approved by the Jiangsu Administrative Committee for Laboratory Animals (Permission number: SYXK-SU-2007–0005).

### Viruses, cells, plasmids and antibodies

Genotype IV NDV strain Herts/33 (from Dr. D. J. Alexander, Animal Health and Veterinary Laboratories Agency, UK) was plaque-purified for three rounds in chicken embryo fibroblasts (CEF). Reverse genetic-derived JS5/05 strain (rJS5/05) has been generated by Hu et al. [18]. Viruses were propagated in 10-day-old specific-pathogen-free (SPF) embryonated chicken eggs. BHK-21 cells (clone BSR-T7/5), generated by Buchholz et al. [19], were grown in Dulbecco’s minimal essential medium (DMEM) with 10 % fetal bovine serum (FBS). The full-length antigenomic cDNA clone of JS5/05 has been previously constructed [18]. Three helper plasmids (pCIZJ1NP, pCIZJ1P and pCIZJ1L) derived from genotype VIId NDV strain ZJ1 were generated by Hu et al. [20]. Monoclonal antibody 6B1 against the HN protein of ZJ1 [21] and goat anti-mouse IgG-FITC (SothernBiotech, Birmingham, USA) were used in the indirect immunofluorescence assay (IFA) as the first and second antibody respectively.

### Construction of the full-length cDNA clone of Herts/33 and virus rescue

Virus RNA was extracted from the infective allantoic fluids using TRIzol Reagent (Life technologies, Carlsbad, CA, US). Briefly, ten overlapped cDNA fragments encompassing the complete anti-genome of the virus were amplified by reverse transcriptase-polymerase chain reaction (RT-PCR). These cDNA fragments were sequentially cloned into the transcription vector TVT (0.0) using the shared restriction sites naturally present in the virus genome, resulting in the full-length cDNA clone of Herts/33 (Fig. 1).

Virus rescue was performed as previously described [18]. BSR-T7/5 cells were transfected with the full-length cDNA clone and three helper plasmids using the FuGENE HD transfection reagent (Roche, Mannheim, Germany). Three days later, the cell culture supernatant was collected and 0.3 ml was inoculated into the allantoic cavities of 10-day-old SPF embryos. Recovery of the virus was confirmed by HA assay. The F and HN genes were sequenced to confirm the virus identity.

### Construction of the recombinant viruses

As illustrated in Fig. 2, the M, F and HN genes of Herts/33 were replaced with the corresponding genes of JS5/05 individually or in combination. Briefly, the M, F and HN genes of JS5/05 were firstly inserted into Herts/33 backbone together using indicated restriction sites and then these genes were replaced by the corresponding Herts/33 genes to generate the recombinant constructs harboring single or two JS5/05 fragments. To retain homologous CT domains of the F and HN proteins, gene fragments including the ectodomains, rather than the entire open reading frames (ORFs), of these two genes, were replaced using the selected restriction sites. In addition, a chimeric virus derived JS5/05 backbone carrying the M, F and HN genes of Herts/33 was also generated as a reference strain. The details for construction of the chimeric full-length cDNA clones are available upon request. The recombinant viruses were rescued as described above. The presence of the M, F and HN genes were confirmed by nucleotide sequencing.

### *In vitro* characterization of the recombinant viruses

The growth kinetics of the parental and recombinant viruses was assessed by a multistep growth assay in DF1 cells. Briefly, each virus was inoculated at a multiplicity of infection (MOI) of 0.001 into DF1 cells that were cultured at 37 °C in DMEM containing 5 % FBS. The supernatant was collected at every 12 h until 60 h p.i. The virus content in the collected samples was titrated as 50 % tissue culture infectious dose (TCID_50_) using the Reed and Muench method [22].

The fusion ability of the recombinant viruses was examined as described by Connaris [23]. Monolayers of DF1 cells grown in 6-well plates were infected with the viruses at MOI of 0.001 and then incubated at 37 °C in DMEM containing 5 % FBS. Twenty-four hours p.i., the supernatant was moved and cells were washed with PBS. The cells were then fixed with methanol and stained with Giemsa method for syncytium observation. The fusion activity was determined by the number of syncytial cells formed compared to the parental virus, rHerts/33.

### Quantification of cell surface expression of HN by FACS analysis

HN is a multifunctional surface glycoprotein of NDV. It possesses the receptor recognition, neuraminidase and fusion promotion activities. Therefore, the cell surface expression of HN of the viruses was quantified using fluorescence activated cell sorter (FACS) method in this study. Briefly, monolayers of DF1 cells grown in flasks were infected with the viruses at MOI of 0.001 and then incubated at 37 °C in DMEM containing 5 % FBS. Twenty-four hours p.i., the supernatant was decanted and the cells were scraped and pelleted by centrifugation. The pelleted cells were fixed with 1 % polyformaldehyde for 1 h. After fixation, the cell pellet was resuspended with phosphate-buffered saline (PBS) and centrifuged for 2 rounds again. Following the 2^nd^ centrifugation, the pellet was incubated with mAb (6B1) against the NDV HN protein at 37 °C for 1 h. Then the cells were washed with PBS and stained with the goat anti-mouse IgG-FITC at 37 °C for 1 h. Finally, the cells were pelleted by centrifugation, washed with PBS and resuspended in a suitable volume of PBS for FACS analysis.

### ICPI and MDT of the recombinant viruses

The pathogenicity of the viruses was determined by measuring ICPI and MDT as described elsewhere [14]. For ICPI, briefly, 0.05 ml of a 1:10 dilution of fresh infective allantoic fluid was injected intracerebally into each of ten 1-day-old SPF chicks. The birds were monitored every 24 h for 8 days. At each observation, the chicks were scored: 0 if normal, 1 if sick and 2 if dead. The ICPI value is the mean score per bird per observation over the 8-day period. For MDT, 0.1 ml of 10-fold dilutions of the infectious allantoic fluid was inoculated into the allantoic cavities of five 10-day-old SPF chicken embryos per dilution and incubated at 37 °C. The eggs were monitored once every 8 h for 7 days, and the time of embryo death was recorded. The MDT is the mean time (h) for the minimum lethal dose of virus to kill all the inoculated embryos.

### Clinicopathological assessment of the recombinant viruses

Groups of ten 4-week-old SPF chickens were intranasally and intraconjunctivally inoculated with 10^6^ 50 % egg infectious dose (EID_50_) of each recombinant virus and two parental viruses. Four birds were mock-inoculated with PBS as the negative control. Three birds per virus-infection group were sacrificed at day 3 and 4 p.i. for gross pathology observation, and the remaining chickens were monitored for mortality. Spleens were collected for tissue section preparation for histopathologic observation. Gross lesion in the spleen was scored based on the following standards: 0 for normal; 2.5 for atrophy without necrosis; 5 for enlarged and mottled; 7.5 for mild to moderate necrotic foci; 10 for extensive and severe necrosis and average scores for three birds were shown. Histologic changes in the spleen were scored as follows: 0 for normal; 2.5 for mild to moderate hyperplasia of lymphocytes; 5 for mild lymphocyte depletion; 7.5 for moderate (<50 %) lymphocyte depletion, histiocytic accumulation and necrosis; 10 for severe (>50 %) lymphocyte depletion, and extensive necrosis, and average values for three chickens were presented.

### Virus load measurement

Groups of fifteen 4-week-old SPF chickens were intranasally and intraconjunctivally inoculated with the chimeric viruses as well as the parental viruses. At 12, 24, 48 and 72 h p.i., spleens from three birds were collected for virus load measurement. Tissue samples were homogenized in PBS containing the antibiotics, and virus titers were measured in CEF. The cleared tissue homogenates were serially 10-fold diluted and inoculated in CEF. At 96 h p.i., cells were fixed with the cold methanol and incubated with the first antibody mAb 6B1 at 37 °C for 1 h to determine the presence of virus. Then the cells were washed with PBS for 3 times and incubated with the secondary antibody goat anti-mouse IgG-FITC at 37 °C for 1 h. Virus titer was determined as TCID_50_ per gram using the Reed and Muench method [22].

### Transcriptional cytokine response

Tissue samples taken at 12, 24 and 48 h p.i. for virus load assay were also processed for cytokine response profiling. Samples collected at 72 h p.i. were excluded because severe pathological changes may lead to indirect effects on cytokine response. Relative expressions of three representative cytokine genes, IFN-β, IL-1β and IL-18, were determined using quantitative real-time PCR (RT-qPCR) and normalized to the housekeeping gene β-actin. Briefly, 1 μg of DNase I-treated total RNA per sample was reverse transcribed into cDNA using RevertAid Premium Reverse Transcriptase (Fermentas, Maryland, USA) at 50 °C for 60 min. The RT-qPCR reaction mixture was consisted of 2 μl of cDNA, 200 nM (final concentration) of each primer and 10 μl of 2 × SYBR Green PCR Master Mix (Takara, Shiga, Japan). PCR reactions were performed in triplicate using the ABI Prism 7300 system (Applied Biosystems, Foster city, CA). The 2^-△△CT^ method was used to determine the fold change of gene expression levels.

### Statistical analysis

The differences of the pathological changes score, cytokine expression levels and virus load among chickens were analyzed using one-way analysis of variance (ANOVA) followed by the least significant difference (LSD) multiple-comparison test. A *p*-value of < 0.05 was considered significant.

## Abbreviations

ANOVA: One-way analysis of variance
CEF: Chicken embryo fibroblasts
CT: Cytoplasmic
DMEM: Dulbecco’s minimal essential medium
EID_50_: 50 % embryo infectious dose
F: Fusion
FACS: Fluorescence activated cell sorter
FBS: Fetal bovine serum
HA: Hemagglutination
HN: Hemagglutinin-neuraminidase
ICPI: Intracerebral pathogenicity index
IFA: Indirect immunofluorescence assay
IFN: Interferon
IL: Interleukin
LSD: Least significant difference
M: Matrix
MDT: Mean death time
MOI: Multiplicity of infection
NDV: Newcastle disease virus
ORF: Open reading frame
PBS: Phosphate-buffered saline
p.i.: post inoculation
RT-PCR: Reverse transcriptase-polymerase chain reaction
RT-qPCR: Quantitative real-time polymerase chain reaction
SPF: Specific-pathogen-free
TCID_50_: 50 % tissue culture infective dose
